# The Parkinsonian Syndrome : Some Points in Diagnosis and Treatment

**Published:** 1924-01

**Authors:** Macdonald Critchley

**Affiliations:** Junior House Physician to the National Hospital for the Paralysed and Epileptic, Queen Square; Late Resident Medical Officer, Hospital for Epilepsy and Paralysis, Maida Vale


### THE PARKINSONIAN SYNDROME; SOME POINTS IN DIAGNOSIS AND TREATMENT.

BY

MACDONALD CRITCHLEY, M.B., CH.B.,

*Junior House Physician to the National Hospital for the Paralysed and Epileptic, Queen Square;*

*Late Resident Medical Officer, Hospital for Epilepsy and Paralysis, Maida Vale.*

---

THIS article, based upon a personal study of seventy-five cases of paralysis agitans, attempts to emphasise some of the less common features of the Parkinsonian syndrome, and also to review its modern treatment. Twenty-five of these cases followed lethargic encephalitis, while fifty were cases of senile paralysis agitans.

### SYMPTOMATOLOGY.

The symptoms common to both types of paralysis agitans are rigidity, weakness and tremor, together with a typical attitude, gait and facies. Any one of these features may be absent, however, necessitating a more detailed study of each symptom.

*Rigidity* usually appears early and gives rise to the sensation of weakness. It is frequently unilateral, but in advanced cases it may involve all four limbs, and later the muscles of the neck, face and jaws. Very rarely the extrinsic muscles of the eye become affected, impeding lateral movements. This phenomenon occurred once only in my series.

The hypertonus occurs in two main types:—

1. The "cog-wheel" or intermittent type, where the resistance to passive movements takes the form of a succession of jerks.

2. The "lead-pipe," a continuous type; this is the more usual variety, and is characterised by an even, sustained resistance to movements in all directions.

The tendon reflexes may be increased in the early stages.

In the post-encephalitic cases the rigidity is often extreme; contractures are frequent owing to the predominating action of the flexors; well-marked involuntary flexion spasms sometimes occur, adding greatly to the patient's discomfort; the facial and ocular muscles tend to be involved more often: for example, one girl was unable to open her eyelids except by raising them with her fingers. Another patient, after looking upwards, found great difficulty in lowering his eyeballs.

The phenomenon of Tonic Innervation, whereby voluntary movements of one group of muscles are immediately resisted by a strong contraction of the opposing set, is another manifestation of extra-pyramidal rigidity occurring after encephalitis.

*Tremor,* which gives the name to paralysis agitans, may however, be absent. It is a fine tremor, but coarser than that of Graves' disease. It starts first in one arm, and spreads to the leg of the same side, afterwards involving the contralateral arm. The lips, tongue and jaw-muscles are affected late in the disease. In the upper limb the tremor is most marked at the metacarpo-phalangeal joints, causing the well-known "pill-rolling" action. The wrist-joints are affected to a lesser degree, and the elbows least of all. The tremor ceases during sleep, and is to some extent under the control of the will. Attempts at voluntary movement temporarily check the tremor; fright, timidity, pleasure and other emotions increase it.

In the post-encephalitic cases the tremor is usually a little different; the maximum movement consists in alternative flexion and extension of the elbow-joints, whilst the fingers scarcely move. The facial muscles are affected early.

The "Parkinsonian Mask" often makes diagnosis very easy. The skin is pale, shiny and greasy with excessive perspiration, and the face in repose is almost expressionless. Voluntary and emotional movements are not markedly affected, however, so that the mask-like face readily lights up with a smile of recognition. It is interesting to recall that the "mask" of paralysis agitans was the only feature that Parkinson omitted to mention in his original monograph.

The attitude of the patient is an extremely valuable diagnostic sign, as it is one of the most constant features of the complaint. The diagnosis of a doubtful case is frequently confirmed when the patient stands upright; he holds himself with his head forward, his shoulders bowed, and his legs slightly flexed at the knee-joints. The arms are bent at the elbows and pronated across the abdomen, with the fingers almost completely extended. The gait is marked by retropulsion and festination, which result from the general rigidity and attitude of flexion.

Rigidity of the muscles of the tongue, lower jaw and lips interferes with articulation and produces a monotonous, somewhat staccato speech with especial defect in the pronunciation of the labials.

Bulbar manifestations not infrequently arise in the course of paralysis agitans; commonest among these is excessive salivation. Incomplete or faulty protrusion of the tongue occurs and dysphagia is frequently present. This last symptom may be simulated by difficulty in mastication and deglutition, due to rigidity and tremor of the masseters and tongue.

The patient's subjective sensations most often take the form of feelings of heat. Consequently he suffers most discomfort during hot weather, and complains of the excessive number of bedclothes at night. Much more rarely the patient complains of cold feelings.

Mental deterioration, taking the form of delusions of persecution, occurred only once among my senile cases, and is apparently an unusual complication. It is common in the post-encephalitic cases, running directly parallel with the extent of physical change. The commonest variety is a progressive dementia characterised by occasional outbursts of disordered conduct and exhibitionism. Temperamental change is very marked both in the primary and in the post-encephalitic cases. In the former group there is an increase in the expression of the emotions, and laughter and tears are readily evoked. The patient, moreover, is frequently deeply introspective and mildly hypochondriacal in the early stages, and it is not uncommon for him to present the doctor at each visit with a closely-written page of his complaints. It is not a matter of surprise, therefore, that many of these early cases are diagnosed as neurasthenics.

The post-encephalitic patient, on the other hand, shows in addition a marked general contentment with himself and his surroundings, a state of mind which is aptly termed "euphoria."

*Senile and symptomatic paralysis agitans.*—The post-encephalitic cases present several other points by which they may be distinguished from the senile cases. Chief in importance are the ocular changes. The commonest sequel of encephalitis lethargica is lack of pupillary reaction on accommodation. This sign is therefore very frequently present in the cases of post-encephalitic Parkinson's disease. Inequality of the pupils cannot be cited as a point in differentiation, as this phenomenon is by no means rare among senile cases.

Next in importance is an ill-sustained or absent convergence of the eyes. The eyes may also show defective conjugate movements causing transient squints and diplopia.

Other abnormalities in the domain of the cranial nerves, such as palsies of the fifth, seventh or ninth nerves, indicate a previous encephalitis.

Whilst the plantar reflex always gives a flexor response in the senile cases, a positive Babinski sign is obtainable in about 50 per cent. of the symptomatic cases.

The age of the patient often assists diagnosis ; cases of paralysis agitans are rare before 45, except when occurring as sequels of encephalitis lethargica.

Biochemical and serological tests, so far as modern knowledge goes, are usually negative.

Thus Wassermann's reaction is negative in the serum, and the figures for the content of nitrogen and calcium in the blood lie within normal limits. The blood count shows no constant alterations. Examination of the cerebro-spinal fluid reveals a negative Wassermann reaction, no excess of globulin or albumin, and no pleocytosis ; Lange's colloidal gold test gives a normal curve. The urine, however, very frequently contains some Fehling-reducing substance in small quantities, of the nature of which I have no knowledge.

### PROGNOSIS.

It is important to diagnose between the primary and post-encephalitic cases of paralysis agitans, as the prognosis of the two conditions differs widely.

Senile paralysis agitans does not constitute a menace to life, and may last for twenty years or more. The progress of the disease is slow, but is usually a steady downward march. Remissions and relapses are both infrequent.

Accurate prognosis in cases following encephalitis is still a matter of uncertainty, but modern observations indicate that there are two main types of encephalitic sequelæ.

1. A group of cases arising at long intervals after the acute encephalitic attack. There is but little physical or mental deterioration, and these cases tend to remain stationary for many months.

2. A large group of cases showing gross mental and physical change. These arise very shortly after the acute attack, and tend to rapid deterioration. Such cases appear to be invariably fatal.

### TREATMENT.

While no specific treatment is known for this disease, or indeed any form of therapy that can be regarded as curative, nevertheless there is no excuse for withholding measures which, if correctly applied, afford the patient a vast amount of relief. Energetic treatment directed towards the various symptoms has the power of alleviating much of the patient's discomfort, and, possibly, of checking the progress of the disease in the senile cases. The main features that will call for relief are the rigidity, the tremor, the bulbar symptoms, insomnia and the uncomfortable sensations of heat.

Firstly, the patient should not be confined to bed, except in the very last stages of the disease. He should be encouraged to exercise and mix freely with others. This serves the double purpose of combating the rigidity and of maintaining the spirits. Confinement to bed hall-marks the patient as an invalid, and may have a disastrous effect upon his morale.

Light, general massage is sometimes of service. It must be given, however, with circumspection, as many patients are extremely intolerant of massage and become profoundly exhausted after each treatment.

Passive movements to the arms and legs are valuable in allaying rigidity and preventing contractures and fixed joints.

Regulated exercises should be ordered and carefully supervised so as to avoid excessive fatigue. Relaxation exercises are of especial value when correctly carried out, and afford the patient a deal of refreshment and comfort.

Galvanism, faradism, sinusoidal baths, and other forms of electrical treatment are not to be recommended.

Perhaps the most valuable form of therapy, however, lies in the daily administration of hot baths. The patient lies for twenty to thirty minutes in water as hot as he can bear, and at the same time voluntarily relaxes all his muscles. The effect in decreasing the hypertonus is immediate; patients have told me that they have at once been able to dry themselves, when previously their rigidity prevented all but the slightest movement. The benefit in cases of insomnia is also very great.

Among the most valuable drugs for this complaint are hyoscine, parathyroid extract, veratrum viride, gelsemium, luminal, the bromides and belladonna.

Hyoscine is probably the most useful drug of all, when administered in suitable doses. As a rule, it is given in much too small amounts, and consequently the results have been disappointing. Improper use has therefore brought discredit upon a valuable drug, as has happened so frequently in modern medicine. The main effect of hyoscine in this disease is upon the rigidity which shows a very rapid response. It moreover checks the excessive salivation and the hyperhidrosis. The effect on the tremor is practically nil; in fact, there may be a temporary increase in the tremor at first, owing to the release of the hypertonus. Hyoscine, therefore, is best prescribed in combination with the bromides, parathyroid, veratrum viride or some other sedative drug. Hyoscine hydrobromide, gr. $\frac{1}{100}$ to $\frac{1}{50}$, is not too large a dose when given by the mouth, and may be repeated two or three times daily.

In obstinate or advanced cases of rigidity it may be necessary to administer hyoscine hypodermically. Thus gr. $\frac{1}{200}$ to $\frac{1}{100}$ injected at bed-time, and again in the morning if necessary, relieves the patient enormously.

Parathyroid extract has been in use in this disease since 1905. The results, however, have been conflicting, due chiefly, I believe, to improper preparations of the gland and also to its inability to improve the rigidity. Nowadays, when one is able to standardise preparations of parathyroid extract, one is forced to admit that it is a drug of value in this disease. Its chief action is a sedative effect on the tremor, combined with a most marked benefit to the patient's comfort and sense of well-being. Patients who have been on parathyroid and then taken off have begged me to put them back again, as they felt so much better when on the drug. There is an appreciable improvement in the appetite, and many patients gain in weight. It is a very valuable adjuvant to treatment with hyoscine.

The bulbar manifestations seem to be particularly amenable to parathyroid medication. Three cases in my series, all showing marked bulbar phenomena (dysphagia, deviation or inability to protrude the tongue, etc.) completely lost these symptoms after a week's treatment with parathyroid. No other drugs were simultaneously employed.

The bromides are of value in allaying tremor and in some measure they relieve insomnia. Their action in this connection is so mild, however, that doses must be employed sufficient to cause depression.

Gelsemium has been tried, particularly in the post-encephalitic cases, but the results have been disappointing.

Another old-fashioned drug which I have thought benefited patients is veratrum viride, given cautiously in three to four minim doses of the tincture. The chief action was upon the tremor.

Phosphorus was formerly an extremely popular drug in this disease, but its use seems to have dropped out of fashion. I have employed it lately in the form of the pil. phosph. gr. 2—4 t.d.s. with but little benefit to the tremor or rigidity. There was a definite general improvement, however, in the patient's malaise and discomfort.

Belladonna may be required to augment the action of hyoscine in cases of profuse salivation.

Strychnine should generally be avoided in paralysis agitans, as it tends to increase the rigidity. As a temporary measure, however, it may be required in cases of extreme bulbar involvement endangering life.

Insomnia when present in this disease is due to inability to relax completely. Excessive tremor or involuntary flexion spasms may also aggravate the sleeplessness. For such conditions veronal or its derivatives are the best hypnotics. Either veronal (gr. 5–7) (diethyl-barbituric acid) or medinal (gr. 5–7) (sod. diethyl-barbituric acid), luminal (gr. 1–2) (phenyl-ethyl barbituric acid) or dial (gr. $1\frac{1}{2}$) (diallyl-barbituric acid) may be administered nightly. These drugs are all very rapid in action ; they afford a refreshing sleep, and produce no untoward effects.

A word of warning must be made against lightly undertaking operative measures upon patients with Parkinson's disease, especially the post-encephalitic cases. One is often tempted to improve deforming contractures around the joints by simple tenotomies or forceful manipulations. The operation, however, appears to be followed by a profound exacerbation of the disease, and the patient is usually left in a much worse condition than before.

